# Decline of *Hesperia ottoe* (Lepidoptera: Hesperiidae) in Northern Tallgrass Prairie Preserves

**DOI:** 10.3390/insects4040663

**Published:** 2013-11-20

**Authors:** Ann B. Swengel, Scott R. Swengel

**Affiliations:** 1 909 Birch Street, Baraboo, WI 53913, USA; E-Mail: aswengel@jvlnet.com

**Keywords:** *Hesperia ottoe*, specialist butterflies, prairie management, butterfly conservation, fire management, butterfly declines, burning, grazing

## Abstract

We counted butterflies on transect surveys during *Hesperia ottoe* flight period in 1988–2011 at tallgrass prairie preserves in four states (Illinois, Iowa, Minnesota, Wisconsin), divided into units cross-referenced to vegetation type and management history. *H. ottoe* occurred only in dry and sand prairie types, and was significantly more abundant in undegraded than semi-degraded prairie, and in discontinuous sod (with numerous unvegetated areas due to bare sand and/or rock outcrops) than in continuous sod. This skipper was significantly more abundant in small sites compared to medium and large sites, even when the analysis was limited to undegraded prairie analyzed separately by sod type. *H. ottoe* was significantly under-represented in year-burn 0 (the first growing season after fire) compared to an expected distribution proportional to survey effort. However, *H. ottoe* was also over-represented in fire-managed units compared to non-fire-managed units. However, by far most units and sites were in fire management and most populations declined to subdetection during this study. Peak abundance post-fire occurred in a later year-burn in discontinuous sod and was much higher than in continuous sod. We also analyze *H. ottoe* status and trend in midwestern prairie preserves by compiling a dataset of our and others’ butterfly surveys from 1974 to 2011. Only 1/9 sites with continuous sod had detectable *H. ottoe* in recent year(s). In discontinuous sod, 2/6 did, with two sites lacking data for the last few years. The number of years *H. ottoe* was still detectable after preservation and the number of years to consistent non-detection were both significantly higher in discontinuous than continuous sod. Both measures of population persistence averaged over twice as long in discontinuous than continuous sod, and correlated negatively with prairie size. The year when consistent non-detection began varied over several decades among sites. Despite the currently urgent need to identify how to manage preserves successfully for *H. ottoe*, such research now needs to be very cautious, because of the extreme fragility of the few remaining populations and the ruggedness of the preserves where *H. ottoe* is still known to occur.

## 1. Introduction

Since European contact in North America, about 99% of tallgrass prairie has been destroyed, primarily by conversion to agriculture [1,2]. As a result, butterflies specialized to prairie became restricted primarily to preserves [3,4,5], including *Hesperia ottoe* W. H. Edwards 1866 (Ottoe skipper). Its conservation has been a subject of study for decades [6,7,8,9,10]. 

In this paper, we analyze transect butterfly surveys during *H. ottoe* flight periods in 1988–2011 at tallgrass prairie preserves in four states (Illinois, Iowa, Minnesota, Wisconsin). These surveys were cross-referenced to vegetation type and management history, with survey effort (time and distance spent surveying) also tracked. As a result, it is possible to analyze skipper abundance in relation to vegetative and management characteristics. These results should be useful for understanding this skipper’s habitat preferences and management responses.

We also use a dataset of our and others’ butterfly surveys as assembled in Schlicht *et al.* [11] and Swengel *et al.* [12] to analyze the status and trend of *H. ottoe* in prairie preserves in the Midwest (Illinois, Iowa, Minnesota, and Wisconsin). Schlicht *et al.* [11] reported that during 1993–1996, two teams (Schlicht and Swengels) happened to survey the same Minnesota prairies in the same seasonal timing in the same years, but without any coordination of sites, transect routes, survey methods, and dates between teams. Strong covariance occurred in abundance indices for individual butterfly species between the two teams. The validation test was necessarily weaker between Saunders’ [13] and Swengels’ surveys in Iowa during 1993–1994 because of less overlap in site and date [12]. However, these comparisons also indicated a positive relationship between teams’ datasets. These analyses validate assembling multiple survey datasets together to examine patterns of butterfly abundance.

The benefit of the multi-team dataset is more coverage of more sites over more years, to provide a more complete assessment of the species’ status and trend. However, because of differences among teams in survey methods, we did not analyze vegetative and management variables in this dataset. Furthermore, because of difficulties in detection and identification of skippers (Hesperiidae), this analysis is a relative assessment. That is, non-detection indicates a drop in relative abundance from a reliably detectable population to a “subdetectable” (undetected) status, which includes both undetected presence as well as absence of a population. It is not readily possible to distinguish between undetected presence and absence in this dataset. Nonetheless, distinguishing between reliably detectable and subdetectable populations is also valuable. Only when populations are reliably detectable can they be studied, monitored, and conserved effectively. We use the multi-team dataset to describe patterns of population persistence and decline in midwestern prairie preserves relative to site characteristics. These results are useful for developing effective conservation strategies for *H. ottoe*.

## 2. Methods

### 2.1. Swengel Surveys

**Figure A1:**
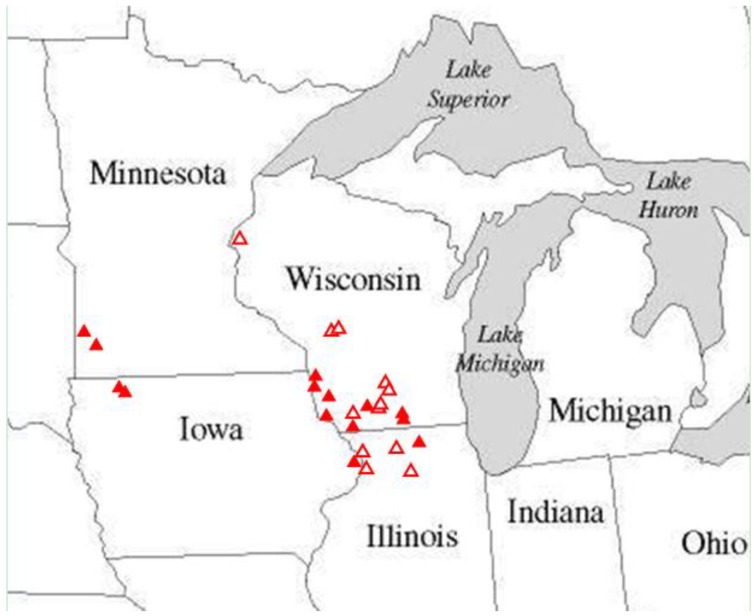
Map of *H. Ottoe* sites analyzed in this study shown as solid triangles, (cf. Appendix Table A1) and sites of plausible habitat shown as open triangles (cf. Table 2).

During 1988–2011, we conducted butterfly transect surveys in prairies (primarily preserves) in Illinois, Iowa, Minnesota, and Wisconsin along similar routes within each site each visit (similar to Pollard [14]), as described in Swengel [15,16,17] (Appendix Figure A1, Table A1). Walking at a slow pace (about 2 km/h) on parallel routes 5–10 m apart, we counted all adult butterflies observed ahead and to the sides, to the limit at which an individual could be identified, possibly with the aid of binoculars after detection, and tracked. For *H. ottoe*, we recorded behavior of each individual, including species of plant nectared or oviposited on. We designated a new sampling unit whenever the habitat along the route varied by management (type and/or years since last treatment), vegetation type (wet, wet-mesic, mesic, dry-mesic, dry, “extra” dry sand), vegetative quality based on amount of brush and diversity and abundance of native and exotic flora (undegraded, semi-degraded, degraded), and/or estimated macrosite canopy (grassland <10%, open savanna 10%–24%, closed savanna 25%–49%, forest opening 50%–75%). Routes crossed rather than followed ecotones and management boundaries to reduce edge effects, and were designed to minimize number of unit changes during the survey while covering representative areas of the site. Classifications of prairie vegetation, management, and size followed Curtis [1], Iowa State Preserves Board [18], Iowa Department of Natural Resources [19], Wendt [20], The Nature Conservancy [21,22], Wisconsin Chapter of The Nature Conservancy [23], Minnesota Department of Natural Resources [24], and Meyer [25]. We recorded temperature, wind speed, percent cloud cover, percent time sun was shining, route distance, and time spent surveying separately for each unit. Surveys occurred during a wide range of times of day and weather, occasionally in intermittent light drizzle, so long as butterfly activity was apparent, but not in continuous rain.

A unit’s management was coded based on the management history observed or evident during the study. Sites managed with fire were typically burned in a rotation of units burned in a different year over 2–5 years, possibly with some mowing or haying or hand-cutting of brush also. Sites with no active management conducted or otherwise evident during the study were categorized as “idle” (non-managed). Early in the study, Hogback was a non-conserved site with continuous moderate dairy grazing at about 3–6 AUM/ha/y (animal use months per hectare per year) (animal use month = 500 kg of cattle for 1 month) but grazing did not occur after 1997 following conservation acquisition. Non-broadcast managements (e.g., hand-cutting of brush) were counted as a treatment only in years when substantial alteration of vegetation occurred. Management year-class (years since last treatment) was coded as 0 years (<1 year) since last treatment, 1 (≥1 but <2 years ago), 2, *etc*. A fire year-class (“year-burn”) of 0 signifies the unit was burned in the cool season (fall or spring, usually the latter) since the last growing season. Thus, a fire after summer 1987 and before summer 1988 is said here to have been burned in 1988 (before the growing season). For units managed with more than one type, management age class was tracked separately for each management type. 

To compare relative abundance among units, we calculated rates of observation as total individuals per total survey time in each unit. For long-term population monitoring, we identified the peak survey per year at each site (standardized to survey time) to represent the butterfly’s abundance at a site, if during the main flight period that year. One survey during main flight period has been adequate for producing representative indices for comparisons of relative abundance within and among sites [11,26,27].

### 2.2. Other Datasets

Schlicht *et al.* [11] and Swengel *et al.* [12] assembled prairie butterfly survey datasets available from Dana [6], research reports by Gerald Selby (1988–1990 and 2003–2005 in Minnesota) and Dennis Schlicht (1993–1997 in Minnesota) posted on the Internet, and Saunders [13] for Iowa 1993–1994, and unpublished results provided by Frank Olsen (Iowa 2004–2008) and Robert Dana (2006–2007). Additional 2011 data for Wisconsin came from Mike Reese [28]. From these datasets and the Swengel surveys, we identified sites where *H. ottoe* had ever been recorded, and assembled time series of detection-nondetection per site per year for as many years as possible.

### 2.3. Statistical Analysis

All analyses were done with ABstat 7.20 software [29]. Statistical significance was set at *p* < 0.05. All statistical tests in this study are non-parametric because they do not require data to be distributed normally. The Spearman rank correlation was used for all correlations, the Wilcoxon signed ranks test for comparisons between paired samples, Mann-Whitney U test for comparisons between unequal samples, and the Chi Square Goodness of Fit test to examine skewing compared to distribution proportional to survey effort.

## 3. Results and Discussion

### 3.1. Habitat Characteristics

In the Swengel dataset (Table 1), *H. ottoe* occurred only in dry upland and sand prairie. All Illinois and Wisconsin sites in this study (Table 2) contained only those prairie types. The Iowa and Minnesota sites (Table 2) contained both lowland and upland prairie. The few records there in the Swengel dataset and all records attributable to a vegetative type in the other datasets occurred only in upland prairie. In the Swengel dataset, *H. ottoe* was significantly more abundant in undegraded prairie (Table 1), and all sites where we found *H. ottoe* contained undegraded dry or sand prairie. *H. ottoe* was also significantly more abundant in sites with discontinuous sod (with numerous unvegetated areas due to bare sand and/or rock outcrops) than in continuous sod (with few or no small bare spots and/or rocks). Small sites had higher *H. ottoe* abundance compared to medium and large sites. When the sample was limited to high-quality prairie analyzed separately by sod type, the preference for small sites persisted, significantly so for discontinuous sod, which had the greater range of site sizes. Because most populations in this study declined to subdetectability either before or during Swengel surveys (Table 2), habitat analysis (Table 1) was limited to extant sites only in Wisconsin in the years the species was still detectable, in order to make any habitat preferences more identifiable statistically. We recorded no *H. ottoe* at a number of other sites appearing to contain appropriate vegetation (Table 3). 

**Table 1:** Mean ± SD of *H. ottoe* individuals per hr in Wisconsin on unit surveys in the Swengel dataset. Analysis only includes years at each site before subdetection occurred (if subdetection occurred). Within each vegetative characteristic, variates sharing any of the same letters are not statistically different by the Mann-Whitney U test (two-tailed *p* < 0.05).

Prairie type	N Unit surveys	Mean observation rate	SD	Statistical grouping
Prairie type				
dry prairie	549	4.58	13.31	A
“extra dry” sand prairie	71	2.74	14.08	A
Prairie quality				
degraded	40	0.43	1.06	AB
semi-degraded	241	1.81	6.49	B
undegraded	340	6.64	16.93	A
Type of sod ^1^				
continuous	385	2.20	7.26	B
discontinuous	236	7.90	19.16	A
Prairie size ^2^				
Small	107	14.29	24.71	A
medium	377	2.01	6.65	B
large	137	3.10	11.20	B
Prairie size ^2^ limited to high-quality continuous sod				
small	18	7.49	14.72	A
medium	168	2.21	6.46	A
Prairie size ^2^ limited to high-quality discontinuous sod				
small	65	20.27	29.07	A
medium	9	5.20	7.84	AB
large	80	4.48	14.33	B
Management				
idling	38	1.17	2.90	AB
grazing	96	0.49	1.13	B
manual cutting	5	0.00	0.00	AB
fire^+^mow/graze	69	2.45	8.00	AB
Fire	413	5.94	15.83	A

^1^ Discontinuous sod has numerous unvegetated areas due to bare sand and/or rock outcrops; continuous sod has few or no small bare spots and/or rocks. ^2^ Small (1.2–1.6 ha), medium (8–20 ha), large (32–38 ha).

**Figure 1:**
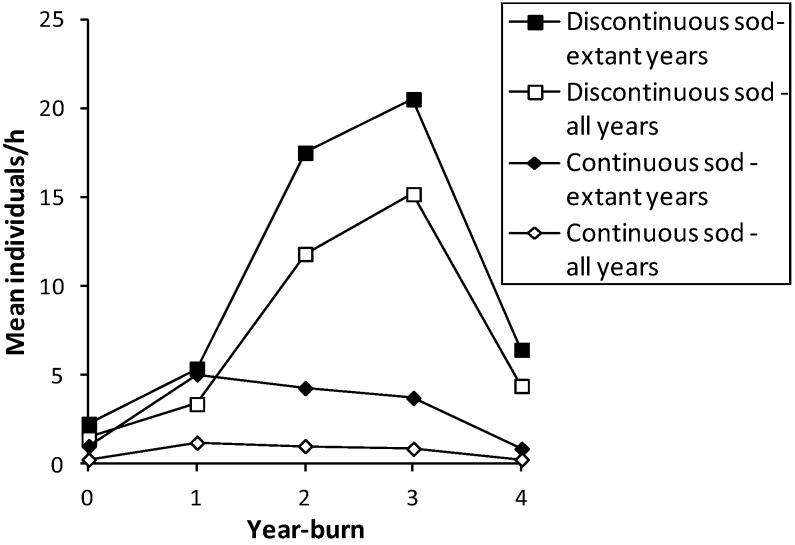
Mean abundance (individuals/h per unit survey) of *H. ottoe* in each year-burn after fire in all survey years before subdetection at each site in the Swengel dataset (see Table 2), by continuous or discontinuous sod.

**Table 2:** Study sites where *H. ottoe* was recorded in this multi-team dataset through 2011. This table identifies the last known year the species was detected (it may not have been detected in all years visited prior to this year), and the first and last year and number of years when all visits had no detection (called “subdetection”).

Site	State	Year preserved	Last year detected	N years detected	1st year subdetectable	N years to subdetection	Year of last survey	N years of subdetection
Continuous sod								
Cayler ^1^	IA	1971	1974	3	1980	9	2005	8
Haffner	IA	1976	1989	13	1994	18	2005	4
Harlem Hill	IL	1973	1993	20	1994	21	1997	3
Hole-in-the- Mountain (new)	MN	1990	1991	1	1992	2	2006	6
Hole-in-the- Mountain (old)	MN	1978	1995	17	1996	18	2007	4
Prairie Coteau	MN	1986	1995	9	1996	10	2007	6
Hardscrabble ^2^	WI	1997			2006	9	2011	4
Hogback	WI	1997	2011	14			2011	0
Muralt Bluff	WI	1977	1997	20	1998	21	2011	14
Oliver	WI	1962	1992	30	1993	31	2011	19
Discontinuous sod								
Manikowski	IA	1985	2008	23			2008	0
Battle Bluff	WI	1983	2011	28			2011	0
Dewey Heights	WI	1952	2007	55	2008	56	2011	4
N Rush Creek	WI	1981	2007	26	2008	27	2008	1
Rush Creek	WI	1981	2011	30			2011	0
Spring Green ^3^	WI	1972	1996	24	1997	25	2011	6

^1^ Dennis Schlicht, pers. comm. ^2^ Species reported as occurring in site in Meyer [25]; not found on surveys in this study. ^3^ Spring Green West (west of Highway 23) was surveyed only in 1993–1995, with one individual found in 1994. Because of inadequate and outdated surveying, this site is not analyzed.

**Table 3:** Sites of plausible habitat in Illinois and Wisconsin for *H. ottoe* surveyed during its flight period in 173.6 h and 403.8 km of survey effort, but with no records of the species in this study. See also Swengel and Swengel [8] for surveying at numerous sites of plausible habitat in Iowa, Minnesota, and North Dakota, but with no. *H. ottoe* recorded.

Site	State	Years surveyed	Latitude	Longitude	Size (ha)
Ayers Sand Prairie	IL	1991–1993	42.06	90.10	47
Bicentennial Prairie	IL	1991–1993	42.13	89.20	3
Nachusa Prairie	IL	1991–1996	41.88	89.35	162
Thomson-Fulton Prairie	IL	1991–1993	41.92	90.11	15
Bauer-Brockway Barrens	WI	1991–2012	44.29	90.75	97
Black Earth Rettenmund Prairie	WI	1990–1993	43.14	89.77	4
Blue River Cactus Flats	WI	1991–1993	43.15	90.53	36
Crex Meadows	WI	1991–2012	45.88	92.60	7000
Dike 17	WI	1990–2012	44.31	90.56	4
Schluckebier Prairie	WI	1991–1993	43.29	89.79	9
Thomson	WI	1990–2012	42.98	89.83	40
Thousand's II	WI	1990–2012	42.98	89.84	2

*H. ottoe* nectared on a wide variety of primarily native flowers (Table 4). We observed oviposition (*i.e.*, confirmed an egg was laid) on a blade of *Schizachyrium scoparium* (little bluestem) three times. We observed oviposition behavior but did not confirm an egg got laid on the underside of *Andropogon gerardii* (big bluestem), on little bluestem, and on a big bluestem blade and adjacent forb foliage.

The significantly higher numbers of *H. ottoe* in undegraded prairie vegetation (Table 1) confirms its categorization at the highest level of prairie dependence [30]. On surveys in 2004–2005 at two conserved sites and adjacent private land in western Iowa, *H. ottoe* significantly related positively with warm-season (native) grasses and bare ground, and negatively with cool-season (primarily non-native) grasses and litter depth [31]. Others have noted the association of *H. ottoe* with high-quality native prairie with short or sparse grass and often some bare areas [32,33,34]. However, the species’ larval food plants are common native prairie grasses [6,30,35] and its nectar sources are widely occurring prairie flowers (Table 4; [6,8]). Undegraded dry or upland prairie, often rocky or sandy, that *H. ottoe* associates with (Table 1; 30) is more widely occurring (even as an intact undegraded prairie flora) than *H. ottoe* (Table 2 and Table 3). Discontinuous sod affords some protection from lethal heat during a fire since the bare sand and/or rock outcrops may prevent some areas from combusting, as evidenced by significantly higher numbers in year-burn 0 in discontinuous sod (Figure 1). As a result, it is unclear whether the significantly higher abundance in discontinuous than continuous sod (Table 1) relates to a vegetative preference or to differential fire mortality.

*H. ottoe* abundance had a negative relationship to prairie size (Table 1) and both measures of population persistence correlated negatively with prairie size (Table 5). This is counter to metapopulation theory that population incidence and abundance should increase in larger and more connected habitat patches [37,38]. Although there was not a significant difference in prairie size between continuous and discontinuous sites, the continuous-sod sites averaged more than double the size of the discontinuous-sod sites (Table 6), and *H. ottoe* was significantly more abundant in discontinuous than continuous sod (Table 1). However, when controlling for sod type, the significant negative relationship to patch size persisted in discontinuous sod (Table 1). We are unable to identify a mechanism for smaller site size to benefit *H. ottoe*. Instead, it appears that patch size has a synergistic relationship with other unfavorable factors, such as tall thick turf and management (discussed below).

### 3.2. Fire Management

*H. ottoe* was significantly more abundant in fire-managed sites than in the grazed site (Hogback), but otherwise management did not produce significant effects (Table 1). *H. ottoe* was under-represented in year-burn 0 compared to an expected distribution proportional to amount of survey effort there (Table 7). However, *H. ottoe* was also over-represented in units ever fire-managed compared to non-fire-managed units (Table 8). When limiting the sample to extant years only, *H. ottoe* was significantly more abundant in discontinuous than continuous sod in both year-burn 0 and year-burn 2+ (Figure 1; Mann-Whitney U tests of unit surveys). When including all survey years, this pattern persisted significantly for year-burn 2+ and nearly so (*p* < 0.06) in year-burn 0. In pair-wise tests within each sod type, year-burns 0, 1, and 2+ were not significantly different from one another in skipper abundance. No tests of abundance were significant when year-burn 2+ was subdivided into year-burns 2, 3, and 4+. However, year of peak mean abundance post-fire occurred later in discontinuous sod (year-burn 3) and was much higher than in continuous sod (year-burn 1) (Figure 1). In discontinuous sod, year-burn 0 averaged a lower abundance than in all subsequent year-burns, but in continuous sod, both the soonest and longest since fire had similarly lowest average abundances (Figure 1).

*H. ottoe* was significantly under-represented in year-burn 0 (Table 7, Figure 1) but also declined in later years of the fire rotation (Figure 1) and was over-represented in fire-managed units compared to non-fire-managed units (Table 1 and Table 8). Likewise, in Vogel *et al.*’s [31] study, *H. ottoe* abundance was significantly higher in burn-only units (1–3 fires during 2000–2004) and significantly lower in both cattle-graze-only (light and rotational) and burned+grazed units (1–4 fires during 1997–2004). In Vogel *et al.* [39], *H. ottoe* peaked in abundance 30 months after fire in highly variable counts plotted out to a maximum of 60 months. 

**Figure 2:**
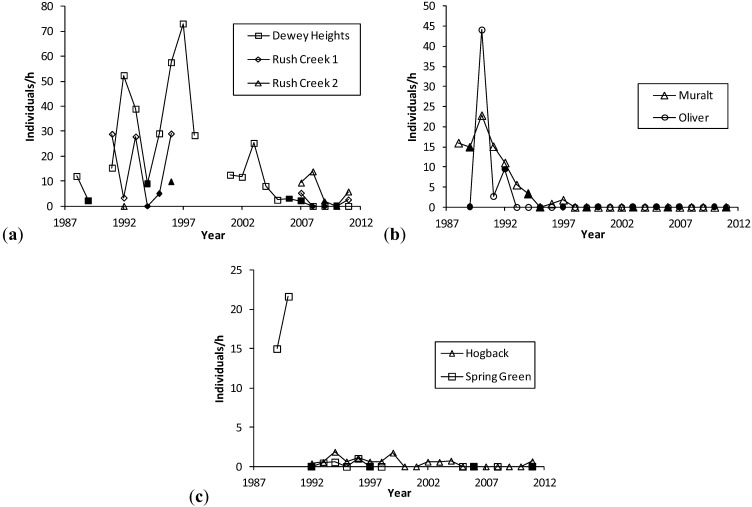
(**a**), (**b**), (**c**). *H. ottoe* individuals per h per year, by site in the Swengel dataset. Dark markers indicate a fire in part or all of core *H. ottoe* habitat in the cool season preceding that summer's survey. The Spring Green Survey in 1991 had *H. ottoe* but occurred at end of flight period and is not graphed here.

However, as most populations declined to subdetection in fire-managed sites (Table 2), these results do not endorse burning as an effective long-term management for this skipper. *H. ottoe* often persisted for some years after the start of fire management, which was usually rotational and partial except that most or all of small sites were more likely to be burned at once during our surveys (e.g., Oliver and Dewey Heights). After a substantial fire in a core area (Oliver and Spring Green in 1992, Muralt Bluff in 1994, Dewey Heights in 2006–2007) the population still existed but then did not recover and became subdetectable within a few years, before the core area burned again (Figure 2). Battle Bluff, one of the few extant sites, has a permanent non-fire refugium in core habitat, according to management staff [40] because the area is too steep for managers to work in safely. Likewise, specialist butterfly abundance was significantly higher in fire-managed sites when they contained a permanent non-fire refugium in core habitat [12,41], which might have unintensive management if needed to control brush and weeds (e.g., mowing problems areas). 

In one experiment, Dana [6] placed reared *H. ottoe* larvae in enclosures in spring. Adult emergences were significantly higher in unburned plots than in plots subsequently burned, with no significant difference between medium and heavy fuel or early and late spring burns. In the next experiment, adult emergences from stocked larvae were significantly lower in plots with moderate fuel that were subsequently burned compared to both burns with light fuel and unburned controls, and in late spring compared to early spring for both fuel loads. Dana [6] concluded that the prior experiment was flawed and based on the latter experiment, that early spring fires in low to moderate fuels typical of dry prairie have mortality too low for concern, while late spring fires of moderate to heavy fuel could be devastating. However, *H. ottoe* has declined and disappeared greatly in burned sites (including Dana’s study site), much more so than that latter experiment would predict. Dana’s [6] earlier experiment (where early spring burns were not more favorable than late spring ones) may be as representative of preserve conditions as the later experiment. Alternatively, a sufficient number of fires may have occurred in these sites in the most unfavorable circumstances (later and heavier fuel conditions). Also, as discussed by Dana [6], ongoing fire management can result in grasses increasing and becoming thicker and taller, leading to less favorable grassland structure for *H. ottoe* as well as heavier fuels.

**Table 4:** N nectar visits by *H. ottoe* during 1988-2012, by Wisconsin site and sex (m = male, f = female, u = unknown). Botanical nomenclature follows Cochrane and Iltis [36]. * = observed in late 1980s; not quantified.

Site Code ^1^	B	Dewey			Hog		Muralt		Oliver			Rush			Spring			Total
Nectar plant	m	m	f	u	m	f	m	f	m	f	u	m	f	u	m	f	u	
*Amorpha canescens* (leadplant)							1									1		2
*Asclepias syriaca*												2	1					3
(common milkweed)																		
*Asclepias tuberosa*		2																2
(butterfly weed)																		
*Asclepias viridiflora*	1	4	2															7
(green milkweed)																		
*Carduus acanthoides*					3			1										4
(welted thistle)																		
*Cirsium hillii* (Hill’s thistle)					4	1	1	1										7
*Cirsium* (thistle)																	1	1
*Coreopsis palmata*	2	10	1						3			21	1					38
(prairie coreopsis)																		
*Dalea purpurea*		4													1	2		7
(purple prairie clover)																		
*Erigeron strigosus*							1									1		2
(daisy fleabane)																		
*Euphorbia corollata*		1																1
(flowering spurge)																		
*Liatris cylindracea*							5	5	2	3	1	3						19
(dwarf blazingstar)																		
*Monarda fistulosa*	1	39	17	3	1		4	3										68
(wild bergamot)																		
*Opuntia* (prickly pear)															*			*
*Pycnanthemum*						2												2
(mountain mint)																		
*Ratibida pinnata*		1	1				1											3
(yellow coneflower)																		
*Rudbeckia hirta*			3				4	1										8
(black-eyed Susan)																		
*Silphium integrifolium*									2									2
(rosinweed)																		
*Silphium laciniatum*														1				1
(compass plant)																		
*Solidago ptarmicoides*										1								1
(upland white aster)																		
*Solidago rigida* (stiff goldenrod)							1	3										4
*Tephrosia virginiana* (goat’s rue)															1			1
*Verbena stricta* (hoary vervain)		1					1											2
Total nectar records	4	62	24	3	8	3	19	14	7	4	1	26	2	1	2	4	1	185

^1^ B = Battle Bluff, Dewey = Dewey Heights, Hog = Hogback, Muralt = Muralt Bluff, Rush = Rush Creek (including North Rush Creek), Spring = Spring Green (main preserve).

**Table 5:** Spearman rank correlation coefficients (r) of patch size and year preserved with N years detected and N years to subdetection, by sod type (continuous or discontinuous), as in Table 2.

Site traits	Continuous sod				Discontinuous sod	
	N sites	r	*p*	N sites	r	*p*
Patch size						
N years detected	9	−0.705	<**0.05**	6	−0.261	>0.10
N years to subdetection	9	−0.540	>0.10	3	−0.500	-
Year preserved						
N years detected	9	−0.435	>0.10	6	−0.551	>0.10
N years to subdetection	9	−0.633	<0.07	3	−0.500	-

**Table 6:** Mean ± SD, median, and range of year preserved and prairie size (ha), by continuous or discontinuous sod as in Table 2. Mann-Whitney U test two-tailed *p* > 0.20 for both.

Site traits	N sites	mean	SD	median	Range
Year preserved					
Continuous	10	1981	11.5	1978	1962–1997
Discontinuous	6	1976	12.4	1981	1952–1985
Prairie size (ha)					
Continuous	10	57.1	62.9	20.2	1.6–157.9
Discontinuous	6	22.5	16.2	24.3	1.2–38.5

**Table 7:** Number of *H. ottoe* individuals observed in the Swengel dataset by whether unit surveys were in the first growing season after burning or not, and expected individuals based on proportion of effort surveying in each sample, only at sites where the species was ever recorded in this dataset. *p* = 0.0000 in both Chi Square Goodness of Fit tests.

Year-burn status	N observed	Time surveying			Distance surveying		
		h	%	N expected	km	%	N expected
Year-burn 0	33	32.69	15.7	105	63.21	17.8	119
Not year-burn 0	637	175.28	84.3	565	291.59	82.2	551

**Table 8:** Number of *H. ottoe* individuals observed in Swengel dataset by whether unit surveys were in fire management or not, and expected individuals based on proportion of effort surveying in each sample, only at sites where the species was ever recorded in this dataset. *p* = 0.0000 in both Chi Square Goodness of Fit tests.

Management	N observed	Time surveying			Distance surveying		
		h	%	N expected	km	%	N expected
Fire	622	148.54	71.2	477	273.77	77.2	517
Non-fire	48	60.20	28.8	193	80.95	22.8	153

Vogel *et al.* [39] identified the direct effect of fire (mortality) as a stronger statistical effect on *H. ottoe* abundance than indirect (vegetative) effects. This agrees with the implication of direct mortality in our study because of the significantly lower abundance in year-burn 0 in continuous than discontinuous sod, which affords some firebreak (Figure 1). However, Vogel *et al.* [31,39] as well as Dana [6] studied *H. ottoe* early in the conservation management history of their study sites, well before the median half-life of *H. ottoe* populations in preserves with discontinuous sod (27 years) and continuous sod (14–18 years), respectively (Table 9). Our study suggests that later in the fire history at a preserve, the indirect (vegetative) effects may be more decisive in population outcome because subdetection began in a year when fire did not occur in core habitat (Figure 2).

**Table 9:** Analysis of population detectability after preservation based on Table 2: N sites in samples and mean, median, and SE of N years (detected or to subdetection), and two-tailed *p* for Mann-Whitney U test between continuous and discontinuous sod.

Sod type	N years detected					N years to subdetection				
	Sites	mean	median	SE	*p*	Sites	mean	median	SE	*p*
Continuous	9	14.1	14	3.01	0.008 ^1^	9	15.4	18	2.91	0.042
Discontinuous	6	31.0	27	6.91		3	36.0	27	10.02	

^1^ *p* = 0.011 if Manikowski is excluded.

In Vogel *et al.* [31], burn-only units had significantly higher warm season (native) grass cover and bare ground (Table 3: mean 20.49%) and lower litter and exotic forbs, while the other two management types had significantly higher cool season (primarily non-native) grass cover. No pre-treatment measurements were available, as is often the case in many conservation management studies, including all sites but Hogback in this study. Without pre-treatment data, it is not possible to identify what floristic characteristics are a response to conservation management, as opposed to a skewing of conservation management decisions based on pre-existing floristic characteristics. That is, fire-only management may be focused on flora that was higher quality upon preservation, while grazing may be focused on areas that were more degraded upon preservation. Thus, differences among plots in flora may not be attributable to conservation management type but instead to conservation preferences in what managements should be implemented in what kinds of flora.

### 3.3. Other Management Types

Idling also did not appear favorable for *H. ottoe* (Table 1). Sites often have brush or weeds at the time of idling and if unaddressed by management, the native floristic quality *H. ottoe* prefers (Table 1) will deteriorate. The small sample for idling could be skewed toward areas deemed lower priority for conservation while the fire-managed sample may be skewed toward areas of higher quality vegetation of higher priority to conservation managers. However, idling could also be unfavorable because it also allows the turf to thicken. As a result, long periods of idling appear more suitable for *H. ottoe* in sites of discontinuous sod such as Battle Bluff, Dewey Heights, and Rush Creek, where the harsh microclimate and rocky soil favor short sparse prairie flora. However, data are not sufficient to determine whether even in those circumstances, idled herbaceous prairie flora is sufficiently compatible with long-term persistence of *H. ottoe*. 

Mowing (either leaving the clippings lie, or removing them as hay) can be relatively favorable for many other prairie-specialist butterflies, including grass-skippers [8,15,17,42]. However, this has not appeared to be a historical management in the *H. ottoe* sites in this study, which do not appear amenable to this agricultural use due to terrain and low vegetative productivity. Theoretically, mowing and brush cutting could be useful for reducing brush and weeds and keeping turf shorter. However, no studies known to us document how *H. ottoe* populations have responded to this management.

Moderate to heavy grazing appears highly detrimental [43], both to the skippers themselves as well as the vegetation they require, and may contribute to the spotty historical distribution of *H. ottoe*. For example, county records are remarkably few in central Iowa and Minnesota [44,45], given the species’ range in all neighboring states as well as Manitoba. Vogel *et al*. [27] found only two individuals in somewhat degraded grazed grassland and only 13 in burned + grazed grassland.

But some *H. ottoe* populations persisted pre-conservation in grazing that was presumably lighter and/or more heterogeneous than typical. Hogback was grazed prior to conservation and had a small population consistently detected each year during 1992−1997 (Figure 2). Upon conservation in 1998, the unit with *H. ottoe* had grazing removed and was idled, with no burning during this study. *H. ottoe* has become less detectable with more time since grazing was removed (Figure 2: detections in 1998−2000, 2002−2004, and 2011). Likewise, McCabe and Post [46] reported very low numbers in grazed areas of the western Dakotas, but found none on the tops of isolated, ungrazed buttes. The two Minnesota sites in this study (Table 2) were grazed prior to conservation [6,47]. Hole-in-the-Mountain had a large *H. ottoe* population at and immediately after conservation [6]; during this period, a substantial area (“new” part in Table 2) later found to be occupied by *H. ottoe* had not been conserved yet. Prairie Coteau had low *H. ottoe* numbers in 1989−1990 in surveys conducted in 1988−1990 [48,49], shortly after the site was conserved in 1986 [50]. No reports are available for the species’ occurrence before and immediately after conservation. Neither site has a record after 1995 ([51]; Table 2). Relatively few data exist on how *H. ottoe* responds to the plethora of possible grazing regimes (species of livestock, intensity and duration of grazing, and frequency if done in rotation). 

### 3.4. Population Status and Trend

**Figure 3:**
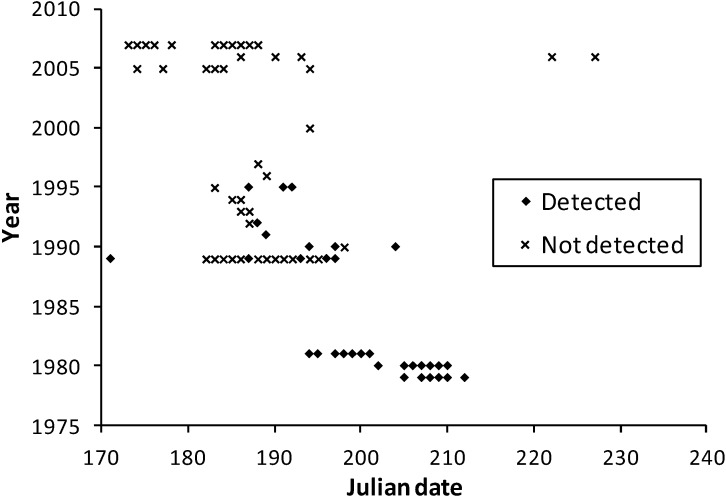
Survey dates in the multi-team dataset for Minnesota at *H. ottoe* sites by whether the species was detected or not.

**Figure 4:**
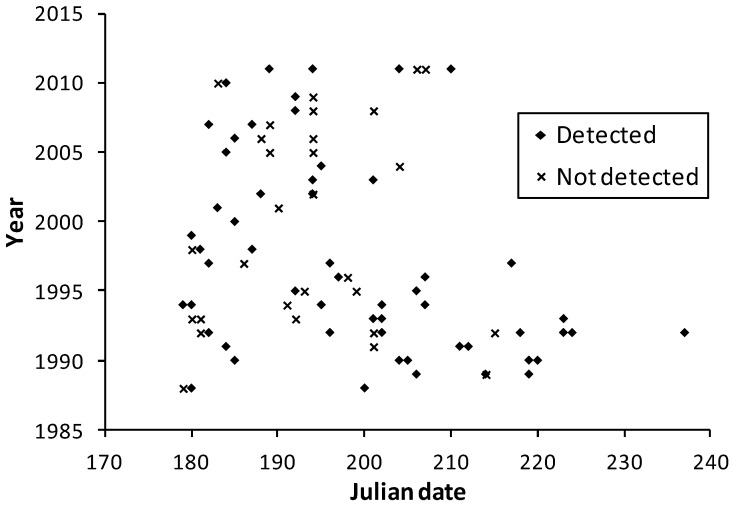
Survey dates in the meta-analysis dataset for Wisconsin at *H. ottoe* sites by whether the species was detected or not.

Observation dates in the multi-team dataset were similar between Minnesota and Wisconsin (Figure 3 and Figure 4), although many more survey dates and longer survey periods per year occurred in Wisconsin. The few records for Illinois (21 July 1993) and Iowa (1 July 1989, 27 July 2005, 24 June 2008) are consistent with the dates in Minnesota and Wisconsin (Figure 3 and Figure 4).

Only 1/9 sites (11%) with continuous sod had detectable *H. ottoe* in recent years (Table 2: Hogback in 2011). In discontinuous sod, 2/6 (33%) did, with two other sites lacking data after 2008. The number of years the species was still detectable after preservation and the number of years to subdetection were both significantly higher in discontinuous than continuous sod (Table 9). Both measures of population persistence averaged more than twice as long in discontinuous than continuous sod. All pair-wise correlations of both measures of population persistence with prairie size and year of preservation were negative (Table 5), one significantly so. Year preserved and prairie size did not differ significantly between continuous and discontinuous sod (Table 6), although sites with continuous sod averaged more than double the size of discontinuous sites. 

Abundance varied greatly within site among years prior to population subdetectability (Figure 2). Years when a core area for *H. ottoe* was burned tended to have abundances at the low end of the range observed in the years immediately before and after these fires. However, low abundances also occurred in other years. The pattern of increasing abundance in the first years following some fires, then a subsequent decrease longer since fire (Figure 1), was apparent in these site time-series (Figure 2). At Dewey Heights (Figure 2), a discontinuous sod site, peak abundances occurred in 1992 and 1996 (year-burn 3) and then fell the following year (year-burn 4). At both Muralt Bluff and Oliver (Figure 2), which are continuous sod sites within 2 km of each other, peak abundance occurred in 1990 (year-burn 1 in the core habitat) and dropped the next year (year-burn 2). At Muralt Bluff, the drop continued each year through 1995 even though fires in the core area did not occur again until 1994−1995. 

Most populations did not become subdetectable in year-burn 0 in core habitat. That is, although numbers were low as a result of the fire, the populations survived the fire but then failed to recover in subsequent years of the fire rotation, as had occurred in prior cycles. For populations detected in 1990, these stalled recoveries and subsequent population failures occurred in the mid-1990s at Muralt Bluff (1996–1997), Oliver (1993), and Spring Green (1993–1996) and in the late 2000s at Dewey Heights (2008). At Hogback, no fire has been observed in the core *H. ottoe* habitat. The population occurred in very low numbers but was detected each year 1992–1999, then became unreliably detectable after that, being recorded only in 2002–2004 and 2011. The site was lightly grazed by cattle through 1997. Then in 1998, the site was preserved and grazing removed.

Continuous sod sites had more rapid declines after preservation and fewer extant populations than discontinuous sod sites (Table 2 and Table 9). *H. ottoe* populations tended to persist longer after preservation where rocky outcrops and/or sparse fuels afforded more firebreak, fires have been relatively less frequent (e.g., Rush Creek), and/or some occupied habitat was in a permanent non-fire refugium (e.g., Battle Bluff) (Table 9). Although *H. ottoe* declines to subdetectability have been occurring for decades on preserves (1980–2008 in Table 2), the year subdetection began exhibits some clustering, especially in the mid-1990s. Climatic variation may interact with management to result in particularly adverse circumstances for skipper breeding success. For example, the widespread very wet growing season in 1993 [13] may have resulted in unfavorably dense and tall grass turfs. Furthermore, the data suggest (non-significantly) that *H. ottoe* may have declined to subdetection more quickly in more recently preserved sites (Table 5). More recently preserved sites may have had more vulnerable populations, or were managed more intensively, or the landscape context has become more hostile in more recent years, adversely affecting new and old preserves alike. 

Since these skippers can have relatively high abundance in recently burned units (Figure 1), Dana [6] recommended a fire regime in which there would always be a unit <3 years since last fire. To reduce risk from fire mortality on population viability, he advised ensuring that core skipper habitat be placed into at least two units, with one of these units burned every two years (*i.e.*, a four-year rotation of these units). Dana [6] also anticipated the possible long-term negative indirect impact of fire (thick tall grass). He noted that episodic brief heavy grazing or rotational haying might be necessary to maintain the appropriate shorter grass structure. The long-term data series in our study bear out this concern, both for the removal of grazing at Hogback and for the longer-term effects of fire at other sites (Figure 2). These long-term effects are particularly challenging because they seem to present a sharp dichotomy: the prior fire rotation supports an expectation of future recoveries and then no recovery at all occurs following the next fire (Figure 2). Thus, by the time the effect sets in (hypothesized here to be an unsuitably tall and dense grass structure), there is very little time to act before population failure. 

## 4. Conclusions

There is an immediate need for short-term results at the start of conservation management, to start the process of identifying more likely beneficial management and to halt any management that registers an immediate unfavorable outcome. However, long-term viability of specialist populations is the ultimate goal of conservation. To determine what managements are more likely to associate with that, it is also necessary to conduct long-term monitoring. Such “prospective” data (tracking populations through time) are included in this study (Figure 2). 

Either an extreme microhabitat in discontinuous sod or a management such as light rotational grazing or other comparable activity (light trampling and brush-cutting) may be needed to maintain the sparse grassland structure suitable for *H. ottoe* with sufficient *H. ottoe* survival in the site in order to sustain a viable population. McCabe [41] identified two management issues at preservation. First, management occurring pre-conservation that might have been favorable (such that the species existed in the site at all) is stopped. Second, a new management is started that might or might not be as favorable as previous management. Unfortunately, whatever positive aspects for *H. ottoe* existed from the management pre-preservation were little studied, because of the rapid conversion to conservation management (primarily fire). Despite the currently urgent need to identify how to manage preserves successfully for *H. ottoe*, such research now needs to be very cautious, because of the extreme fragility of the few remaining populations and the ruggedness of the preserves where *H. ottoe* is still known to occur (Table 2).

## Appendix

**Table A1:** Sites with *H. ottoe* records surveyed in the Swengel dataset during *H. ottoe* flight period, totaling 235.2 h and 402.5 km of survey effort.

Site	State ^1^	Years surveyed	Latitude	Longitude	Size (ha)
Cayler	IA	1994–1996	43.30	95.10	64.8
Haffner	IL	1989, 1994, 1997	43.25	95.05	20.2
Harlem Hill	IL	1993–1997	42.33	89.02	20.2
Hole-in-the-Mountain (new)	MN	1990–1997	44.25	96.30	157.9 ^2^
Hole-in-the-Mountain (old)	MN	1989–1997	44.25	96.30	157.9 ^2^
Prairie Coteau	MN	1992–1997	44.12	96.15	113.4
Battle Bluff	WI	2007–2008,2010	43.46	91.20	8.1
Dewey Heights	WI	1988–89, 1991–98, 2001–11	42.73	91.02	1.22
Hardscrabble	WI	2006–2008	43.22	90.10	4.05
Hogback	WI	1992–2011	43.23	90.87	16.2
Muralt Bluff	WI	1988–2011	42.70	89.50	15.0
Oliver	WI	1989–2011	42.69	89.50	1.62
North Rush Creek	WI	2007–2008	43.37	91.13	38.5 ^2^
Rush Creek	WI	1991–1996, 2007–2011	43.37	91.12	38.5 ^2^
Spring Green	WI	1988–98, 2005–06, 2008, 2011	43.20	90.07	32.4
Spring Green West	WI	1993–1995	43.22	90.10	8.1

^1^ IA = Iowa, IL = Illinois, MN = Minnesota, WI = Wisconsin. ^2^ The same site is broken into two lines in this table to reflect differences in conservation history (see Table 3) or years surveyed.
